# What question are we trying to answer? Embracing causal inference

**DOI:** 10.3389/fvets.2024.1402981

**Published:** 2024-05-21

**Authors:** Jan M. Sargeant, Annette M. O’Connor, David G. Renter, Audrey Ruple

**Affiliations:** ^1^Department of Population Medicine, Ontario Veterinary College, University of Guelph, Guelph, ON, Canada; ^2^Department of Large Animal Clinical Sciences, College of Veterinary Medicine, Michigan State University, East Lansing, MI, United States; ^3^Center for Outcomes Research and Epidemiology, College of Veterinary Medicine, Kansas State University, Manhattan, KS, United States; ^4^Department of Population Health Sciences, VA-MD College of Veterinary Medicine, Virginia Tech, Blacksburg, VA, United States

**Keywords:** causation, observational studies, veterinary, variable selection, confounding

## Abstract

This study summarizes a presentation at the symposium for the Calvin Schwabe Award for Lifetime Achievement in Veterinary Epidemiology and Preventive Medicine, which was awarded to the first author. As epidemiologists, we are taught that “correlation does not imply causation.” While true, identifying causes is a key objective for much of the research that we conduct. There is empirical evidence that veterinary epidemiologists are conducting observational research with the intent to identify causes; many studies include control for confounding variables, and causal language is often used when interpreting study results. Frameworks for studying causes include the articulation of specific hypotheses to be tested, approaches for the selection of variables, methods for statistical estimation of the relationship between the exposure and the outcome, and interpretation of that relationship as causal. When comparing observational studies in veterinary populations to those conducted in human populations, the application of each of these steps differs substantially. The *a priori* identification of exposure–outcome pairs of interest are less common in observational studies in the veterinary literature compared to the human literature, and prior knowledge is used to select confounding variables in most observational studies in human populations, whereas data-driven approaches are the norm in veterinary populations. The consequences of not having a defined exposure–outcome hypotheses of interest and using data-driven analytical approaches include an increased probability of biased results and poor replicability of results. A discussion by the community of researchers on current approaches to studying causes in observational studies in veterinary populations is warranted.

## Introduction

Early in every epidemiology student’s training, they are indoctrinated with the mantra that “correlation/association does not imply causation.” Numerous examples of non-causal associations exist; one such example is the finding that the number of human births over time is correlated (*p* = 0.008) with the number of stork breeding pairs in European countries (1), and yet it would be ludicrous to conclude that storks cause babies. The association is either random or related to the presence of a confounding variable.

There are two main reasons why associations do not imply causation: temporal ambiguity and spurious (non-causal) associations (2). Temporal ambiguity occurs because the temporal sequence of the two correlated variables may not be clear or identifiable. For instance, although stork density and human birth rates are correlated over time in Eastern Europe, the correlation does not address whether stork densities are antecedent or a consequence of human birth rates. Non-causal relationships may also explain apparent associations. These may include confounding factors. For instance, it is plausible that the apparent association between stork density and human birth rates is related to the confounding effects of socioeconomic status; when times are good, people may be more likely to add a child to their family, and there also may be more food waste during good economic times, increasing the number of storks in an area.

From a research perspective, issues of temporal ambiguity and confounding can both be addressed by random allocation to the intervention group (2). For this reason, experiment approaches such as randomized controlled trials are considered the strongest research design for establishing causation. Nonetheless, observational studies are common in veterinary medicine. It may not be ethical or feasible to randomly allocate modifiable risk factors to study subjects, and the necessary sample sizes may be prohibitively expensive, especially for interventions allocated at higher organizational levels, such as pen or herd. In addition, the study populations in observational studies may be more representative of source populations than in experimental trials, and observational settings may better reflect multifactorial disease causation (3). Finally, large observational datasets may exist for animal populations (e.g., medical record systems for companion animals and production databases for food animals), and these may be available for researchers (2).

## Identifying causal associations is a common purpose in observational research

Observational studies may be conducted for several reasons: to estimate a single parameter such as incidence or prevalence, to predict an outcome (e.g., to identify at-risk individuals or populations or for prognostic purposes), to identify possible exposures for further study (exploratory or “hypothesis-generating” studies), or to identify causal relationships (“hypothesis-testing” studies). In observational studies in animal populations, identifying causal relationships is a common purpose; in an evaluation of 200 observational studies in the veterinary literature published between 2020 and 2022, causal wording was used in 86% of the articles (4). Additionally, a further evaluation of 100 randomly selected studies from the Sargeant et al. (4) study found that 70% of the studies did not state that the purpose was prediction and they either discussed the potential for confounding (a causal construct) or conducted multivariable statistics. Therefore, it might reasonably be assumed that the purpose of these 70 studies was to identify causal relationships.

## Comparison of observational approaches to studying causes in the human versus veterinary literature

Ahern proposed a four-step framework for studying causal relationships in human health research (5). The steps include the following: (1) articulating the causal question (identifying exposure: outcome pairing(s) of interest and describing the causal parameter of interest); (2) linking causal and statistical parameters by considering the assumptions under which the exposure groups are equal (identification of confounding variables); (3) estimating the statistical parameter (controlling for confounding); and (4) interpreting the findings as causal effects (theoretical considerations). How are these steps applied in studies in veterinary populations where the intent is to identify causal relationships? Is the approach to identifying causal relationships in the veterinary literature the same as the approach in the human literature? To address these questions, we evaluated the 70 studies (above) where the purpose was assumed to be the identification of causal relationships and compared the results to those from a study by Staerk et al. on observational studies conducted in human populations (6).

Staerk et al. evaluated methodological approaches in 272 observational studies of human populations published in 2019 in four epidemiological journals (Epidemiology, American Journal of Epidemiology, European Journal of Epidemiology, and International Journal of Epidemiology) (6). Staerk et al. distinguished between “hypothesis generating” (exploratory) studies and “hypothesis testing” (causal) studies (6). The definition of causal studies was that the authors defined one or more exposure–outcome pairings of interest, which is the first component of articulating a causal question. Of the 272 observational studies of human populations, 94% included one or more defined exposure–outcome pairings of interest, as compared to 15 of 70 (21%) in observational studies of veterinary populations. This is not a direct comparison because the study of human populations selected articles from four epidemiology journals, whereas the study in veterinary populations did not include any discipline-specific journal restrictions. Nonetheless, it appears that causal studies—or at least the identification of exposure–outcome pairings of interest—are more common in observational studies of human populations. Additionally, only 3 of the 70 studies in the veterinary literature included a statement that the purpose of the study was causal, and none of these explicitly defined the causal parameter of interest (i.e., direct or total causal effect).

The second and third steps in the framework for causal studies involve the identification of confounding variables and the approaches to their control. It is recognized that a preferred approach for the selection of confounding variables is the use of prior knowledge of the underlying causal structure, ideally using Directed Acyclic Graphs (DAGs), with data-driven methods less appropriate to adequately control for confounding (7). Data-driven (algorithm-based) methods for controlling confounding include the use of *p*-values for variable selection (e.g., stepwise selection), methods based on changes in beta-coefficients, and selection of variables to identify individual predictors for inclusion in multivariable model building (univariable screening). Evaluations of the approaches to selecting confounding variables have been conducted on observational studies in human populations published in 2008 (8), 2015 (9), and 2019 (6). In all three of these studies, the observational studies were published in the American Journal of Epidemiology, the European Journal of Epidemiology, Epidemiology, and the International Journal of Epidemiology, all considered to be high-impact journals. The results of these studies and the 70 observational studies in veterinary populations are shown in Table 1. In observational studies of human populations, the use of prior knowledge to select confounding variables has increased over time, from 28 to 73%. Although trends over time were not assessed for observational studies in veterinary populations, prior knowledge was used to select variables in 14% of the studies published in the veterinary literature during approximately the same time period as the study by Staerk et al. (6), reporting that 73% of observational studies in human populations used prior knowledge for variable selection. In the human population studies, the use of data-driven methods to select variables decreased from 37% for studies published in 2008 to 16% for studies published in 2019. However, data-driven methods to select confounding variables are the norm in observational studies of veterinary populations, with these techniques employed in 93% of studies published between 2020 and 2022. The main reason for the differences between observational studies in human versus veterinary populations appears to be the high proportion of studies in veterinary populations where univariable screening and/or *p*-value-based selection approaches are used.

**Table 1:** Variable selection methods for control of confounding in observational studies in human epidemiology journals over time and veterinary populations between 2020 and 2022.

Variable selection method^a^	Human epidemiology journals, 2008 (*N* = 300, results as %)^b^	Human epidemiology journals, 2015 (*N* = 292, results as %)^c^	Human epidemiology journals, 2019 (*N* = 272, results as %)^d^	Causal studies in veterinary populations, 2020–2022 (*N* = 70, results as %)
Prior knowledge	28%	50%	73%	14%
*Prior knowledge using DAGs*	NA	NA	13%	7%
Data-driven methods	37%	24%	16%	93%
*Change in estimate*	15%	12%	7%	7%
*Use of p-values (e.g., stepwise selection)*	29%	5%	4%	69%
*Univariable screening*	NA	9%	4%	76%
Other methods	3%	2%	3%	9%
Not described	35%	37%	16%	6%

^a^Variable selection categories are not mutually exclusive. Therefore, percentages within columns may sum to more than 100%. ^b^Walter and Tiemeier, 2009 (8). ^c^Talbot and Massamba, 2019 (9). ^d^Staerk et al., 2024 (6).

The final step in the framework for causal inference pertains to interpreting the results as causal. One way to consider whether a causal interpretation is appropriate is to consider the guidelines proposed by Sir Bradford Hill (10). These include strength of association, specificity of association, consistency, temporality of exposure and outcome, biological gradient, biological plausibility, coherence with current knowledge, experimental confirmation, and analogy. The application of Hill’s criteria was not accessed in the study by Staerk et al. (6). Based on the information provided in the discussion section of the 70 publications that were deemed to be causal studies of veterinary populations, the concepts provided in Hill’s guidelines were seldom discussed. Although biological plausibility and coherence were routinely addressed, these discussions tended to be framed as general comparisons to the results of other studies without discussing whether these comparisons strengthened or weakened a causal argument for any of the associations identified in the study. A discussion of the temporal sequence of the exposure relative to the outcome was included in seven publications, and the need for experimental confirmation was discussed in four publications. For three studies, the authors explicitly stated that the study design used was not appropriate for causal inference. It should be noted that Sir Bradford Hill did not intend the guidelines to be used as “causal criteria,” and not all of the concepts are necessary or achievable. Ioannidis argues that consistency, temporality, and experimental confirmation are the most relevant concepts for causal inference, although even these are not always possible or straightforward to determine (11). Nonetheless, it appears that the discussion sections in literature from observational studies in veterinary populations are neither strengthening nor disputing causal claims.

## Implications of differences in approaches between causal observational studies in the human versus veterinary literature

The application of the four steps for causal studies in the veterinary literature suggests that there are substantive differences in approaches in the human literature and that inappropriate (or less than ideal) approaches are common in studies of veterinary populations. This then begs the question, “does it matter?.” We argue that it does matter; if the purpose of an observational study is to identify causal associations, then not having one or more defined (and *a priori*) exposure–outcome pairs of interest and using data-driven methods to identify confounding variables may lead to biased results due to inappropriate control of confounding, inappropriate uses of *p*-values, and the use of questionable research practices such as HARKing (hypothesizing after the results are known), p-hacking, and data dredging.

Data-driven methods to identify confounders may be problematic, as a computer algorithm cannot distinguish between confounding variables, colliders, or intervening variables. This can lead to bias in estimating the exposure effect size (6, 12, 13). Another example of inappropriate control of confounding is illustrated by the “Table 2 fallacy.” The Table 2 fallacy refers to the presentation of results from a multivariable model as though each variable can be considered an exposure of interest with the remaining variables corresponding to confounders (14). This interpretation assumes that the causal structure (and therefore the confounding variables that need to be included) is the same for each of the variables in the model, an assumption that may not be true. Table 2 shows that fallacies appear to be common in veterinary medicine. In the 70 observational studies in veterinary populations explored herein, there were four publications where only univariable results were presented (but were categorized as causal because confounding was discussed), four publications where it was not possible to distinguish whether the results represented univariable or multivariable models, and four publications where there were one or no significant results. Of the remaining 58 publications where multivariable results were presented, 54 (93%) included results that could be considered a Table 2 fallacy.

There is a plethora of information on the uses and abuses of *p*-values related to inference about the effect size (i.e., null hypothesis significance testing), and the interested reader is referred to available resources on this topic [for example (15, 16)]. In the context of variable selection for causal studies, there are issues related to p-values and the importance of the effect size. *p*-values do not provide information on the clinical or biological importance of an association (e.g., the effect size that would represent an appreciable benefit or harm of applying an intervention). Additionally, some studies likely are not sufficiently powered to find meaningful differences as statistically significant for multiple variables that were identified as possible exposures *post hoc*. The confidence intervals on an effect size may, therefore, include an association representing a meaningful difference and yet not meet an arbitrary significance cut point for inclusion in a multivariable model.

Not having one or more exposure–outcomes of interest defined *a priori* may lead to the use of techniques involving cherry-picking results or question trolling, such as HARKing and p-hacking. These approaches can lead to biased results (17, 18). These and similar practices may be associated with an increased probability of type I errors. Statistically significant results are more likely to be reported within a manuscript, and studies with statistically significant results are more likely to be published (19). Cherry-picking results or question trolling can lead to type I errors, biased estimates becoming theory, and results for observational studies not being replicable.

## In defense of HARKing

It should, however, be noted that although data-driven approaches to variable selection may lead to biased results, subject-matter knowledge may not always be sufficient to provide clear input to the identification of potentially confounding variables that need to be considered (20). Therefore, *post hoc* data-driven analyses may be of value for moving knowledge in a subject forward (21). However, the analyses should be reported as *post hoc*, and the results should be reported as exploratory. Hollenbeck and Wright refer to this practice as THARKing (Transparently HARKing) (21). However, from the dataset of 70 causal observational studies in veterinary populations, only 3 of the 55 that did not define one or more exposure–outcome pairings of interest reported that their analyses and results were exploratory. Thus, there is considerable room for improvement in the transparency of reporting.

## Discussion

The comparison between observational studies of causal associations conducted in human populations versus veterinary populations highlights some substantive differences in approaches. In particular, approaches to research question formulation and confounding variable selection in studies in the veterinary literature may be prone to providing biased results. If observational studies of causal associations in the veterinary literature are to remain relevant in the broader epidemiological literature, these issues need to be addressed. Short-term solutions, which could be implemented immediately, include clearly describing the purpose of an observational study as causal, exploratory, or predictive. Methods and material sections could be expanded to include a stronger rationale for the identification and control of confounding variables, and ideally a DAG of the hypothesized causal pathways. Discussion sections could be modified to include an explicit discussion of the strength of causal arguments (causal studies), needed research (exploratory studies), or predictive strength of the model (predictive studies). In the longer term, there is a need for epidemiologists conducting observational studies in veterinary populations to discuss the implications of differences in our approach from studies in the human literature and to determine a path forward. Change will require concerted efforts by not only researchers but also mentors of the next generation of researchers, peer-reviewers, and journal editors. In this era of “One Health,” it is time to embrace “One Epidemiology.”

## Data availability statement

The data analyzed in this study is subject to the following licenses/restrictions: anonymized data available for research purposes from the first author upon request. Requests to access these datasets should be directed to JS, sargeanj@uoguelph.ca.

## Author contributions

JS: Conceptualization, Formal analysis, Writing – original draft. AO’C: Conceptualization, Writing – review & editing. DR: Conceptualization, Writing – review & editing. AR: Conceptualization, Writing – review & editing.
